# Cæsarean Section

**Published:** 1883-05

**Authors:** 


### Cæsarean Section.

Prof. Spaeth, of Vienna, performed Cæsarean section and sewed up the uterine wound with five deep and four superficial catgut stitches, largest size of Lister's antiseptic chromic acid ligature. The woman died forty-eight hours after of peritonitis. The autopsy was surprising in its revelations. Every catgut suture in the uterine tissue was found untied and straightened out, while the wound was open and gaping, the lochial discharges having escaped into the peritoneal cavity. The original knots in the catgut had been tied with especial care by Prof. Weinlechner.—*Phil. Med. Times.*

